# Immune Profile in Blood Following Non-convulsive Epileptic Seizures in Rats

**DOI:** 10.3389/fneur.2019.00701

**Published:** 2019-07-02

**Authors:** Una Avdic, Matilda Ahl, Maria Öberg, Christine T. Ekdahl

**Affiliations:** ^1^Inflammation and Stem Cell Therapy Group, Division of Clinical Neurophysiology, Lund University, Lund, Sweden; ^2^Department of Clinical Sciences, Epilepsy Center, Lund University, Lund, Sweden

**Keywords:** non-convulsive status epilepticus, serum, spleen, epilepsy, seizures, peripheral immune response

## Abstract

Non-convulsive status epilepticus (NCSE) is a prolonged epileptic seizure with subtle symptoms that may delay clinical diagnosis. Emerging experimental evidence shows brain pathology and epilepsy development following NCSE. New diagnostic/prognostic tools are therefore needed for earlier and better stratification of treatment. Here we examined whether NCSE initiates a peripheral immune response in blood serum from rats that experienced electrically-induced NCSE. ELISA analysis showed an acute transient increase in serum protein levels including interleukin-6 6 h post-NCSE, similar to the immune reaction in the brain. At 4 weeks post-NCSE, when 75% of rats subjected to NCSE had also developed spontaneous seizures, several immune proteins were altered. In particular, markers associated with microglia, macrophages and antigen presenting cells, such as CD68, MHCII, and galectin-3, were increased and the T-cell marker CD4 was decreased in serum compared to both non-stimulated controls and NCSE rats without spontaneous seizures, without correlation to interictal epileptiform activity. Analyses of serum following intracerebral injection of lipopolysaccharide (LPS) showed an acute increase in interleukin-6, but at 4 weeks unaltered levels of MHCII and galectin-3, an increase in CD8 and CD11b and a decrease in CD68. None of the increased serum protein levels after NCSE or LPS could be confirmed in spleen tissue. Our data identifies the possibility to detect peripheral changes in serum protein levels following NCSE, which may be related to the development of subsequent spontaneous seizures.

## Introduction

Status epilepticus (SE) is a prolonged epileptic seizure with continuous epileptiform brain activity that if not interrupted can develop into a serious medical condition. The most frequent clinical manifestations of SE are convulsive tonic-clonic features, but in 20–40% it may display as altered consciousness and behavior, automatism or subtle motor signs referred to as non-convulsive SE (NCSE) (1–3). Clinically, NCSE can be challenging to diagnose and treatment may be delayed due to this.

The pathophysiological consequences of prolonged NCSE have so far been difficult to predict. Neuroradiological imaging during NCSE in patients may show brain abnormalities such as regional hyperperfusion and reduced diffusion, but most often these changes are resolved within a month (4, 5). However, emerging experimental evidence suggests neurodegeneration, gliosis, and molecular changes in the brain as a result of NCSE induced by electrical stimulation of intraparenchymal electrodes or intracerebral injections of kainic acid (6–8). The inflammatory response initiated within the epileptic focus includes phenotypic cellular changes and increased number of activated microglial cells and astrocytes (7). Possibly, an innate immune reaction in the brain as a result of NCSE leads to activation and recruitment of systemic immune cells, as observed experimentally in a kainic acid model of temporal lobe epilepsy (9). Acute infiltration of CD4^+^ and CD8^+^ T cells into the brain at 24–72 h following a single tonic-clonic seizure has been reported (10, 11), along with long-lasting monocyte and lymphocyte infiltration in both clinical and experimental studies of epilepsy (12). Altered levels of CD8 have been observed in experimental models of SE (13, 14) and infiltrating CD45^+^ leukocytes and CD3^+^ lymphocytes have also been observed within resected temporal lobes from patients with severe therapy-resistant temporal lobe epilepsy (9).

A systemic immune reaction following NCSE may be detected in a blood sample. In rodents with severe convulsive SE, increased levels of circulating CD8^+^ cytotoxic T lymphocytes have been observed in serum (13, 14) and clinical studies describe acute increased levels of pro-inflammatory cytokines, neutrophils and cytotoxic T lymphocytes in serum and plasma from patients after temporal lobe seizures (10, 15, 16). Apart from the blood, peripheral lymphoid organs such as the spleen may also reflect brain inflammation. The spleen plays a central role in recruiting and priming immune cells and recent evidence suggests a link between brain injury and the release of pro-inflammatory cytokines by resident macrophages in the spleen (17). Increased levels of cytokines have also been reported in activated rat splenocytes following epileptic seizures (18).

Here we investigated the peripheral immune response in serum and spleen 6 h, 24 h, 1 w, and 4 w following NCSE, with or without subsequent development of spontaneous seizures. We compare the immune response to serum from rats with intracerebral injection of the bacterial antigen lipopolysaccharide (LPS). LPS is extensively used to induce inflammation in the brain through both peripheral and intra-cerebral administration (19–21) and is known to elicit extensive microglial activation in the brain by mimicking gram negative bacteria (22). We performed intrahippocampal LPS injections to elicit an inflammatory response in the hippocampus (HPC) and compared it to the immune response elicited following NCSE in order to evaluate the specificity of the peripheral immune response.

## Materials and Methods

### Animals

Adult male Sprague-Dawley rats (*n* = 109) weighing between 200 and 250 g were procured from Charles River (Germany). The animals were housed individually in cages with bedding, in 12 h light/dark cycle with *ad libitum* food and water. Procedures were approved by the regional Malmö/Lund committee for experimental animal use and the Swedish board of Agriculture (protocol number M93-14). Animals were used in accordance with the European Community Council Directive (2010/63/EU) and the Swedish Animal Welfare Act (SFS 1988:534) and all experiments were performed in accordance with relevant guidelines and regulations. General health status was monitored daily and all efforts were made to minimize suffering during the experimental study. All surgeries were performed under isoflurane anesthesia. Animals were euthanized if they lost 15% of the original weight. No animals were lost during the study.

### Group Assignments

Rats were randomly divided into four survival groups following electrically-induced SE and corresponding non-stimulated controls: 6 h (SE *n* = 11 and Ctrl *n* = 11), 24 h (SE *n* = 6 and Ctrl *n* = 8), 1 w (SE *n* = 6 and Ctrl *n* = 6), and 4 w (SE *n* = 16 and Ctrl *n* = 12). At 4 w following NCSE, some rats had developed spontaneous recurrent seizures (*n* = 12) while others only displayed acute symptomatic seizures during the first week post-NCSE (*n* = 4).

### Surgeries, Drug Infusions and Electrically-Induced Temporal Status Epilepticus

**Figure 1:**
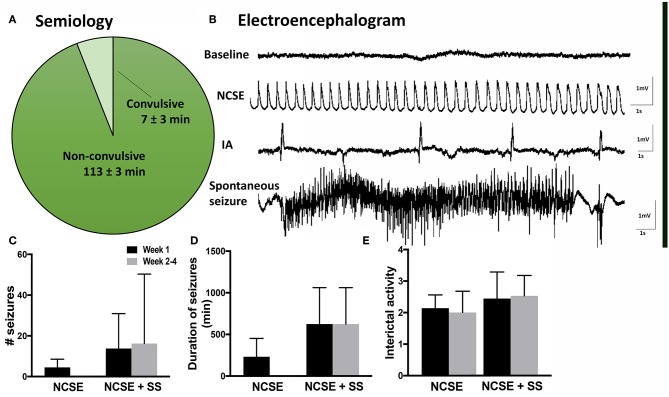
Seizure semiology and electroencephalogram in rats with non-convulsive status epilepticus (NCSE). **(A)** Pie chart showing mean percentage of time the rats exhibit non-convulsive seizure semiology during 2 h of self-sustained NCSE (*n* = 41). **(B)** Representative baseline electroencephalographic (EEG) activity before stimulation, interictal activity, epileptiform activity during self-sustained complex partial NCSE and an example of a spontaneous seizure with an evolving EEG pattern, **(C)** mean number of seizures per rat, **(D)** mean duration of seizures, and **(E)** mean interictal acitivty per animal. Non-convulsive status epilepticus (NCSE), interictal activity (IA).

Animals were anesthetized with 2% isoflurane and implanted with a bipolar insulated stainless steel electrode (Plastics One, Roanoke, VA) into the right ventral CA1/CA3 region of the HPC (coordinates: 4.8 mm posterior and 5.2 mm lateral from bregma; and 6.3 mm ventral from dura, tooth bar set at −3.0 mm) for stimulation and recording. A unipolar electrode was placed between the skull and adjacent muscle to serve as ground electrode. Following a week of recovery after surgery, rats were subjected to electrically-induced temporal NCSE according to previously described protocol (7, 23). Briefly, afterdischarge thresholds were determined for each rat by applying a square wave biphasic pulse (50 Hz) of 1 s train duration at a starting intensity of 10 μA, then increasing with 10 μA every 1 min until a 10 s afterdischarge was evoked. For inducing SE, suprathreshold stimulation was applied for 1 h, with interruptions in the stimulation every 9th minute to record electroencephalographic (EEG) activity for 1 min. Thus, in total rats were stimulated six times with continuous electrical stimulation for 9 min each time, followed by 1 min of EEG recording. After stimulation, most rats developed self-sustained NCSE and subsequently, the ictal EEG activity was recorded for another 2 h and seizure semiology was classified continuously according to Racine's scale. The response in rats during NCSE included mostly non-convulsive features with intermittent convulsive events. Electrode-implanted non-stimulated rats served as controls. Only rats that displayed self-sustained ictal/epileptiform EEG activity for 2 h (Figure 1) in the temporal lobe and mainly partial seizure semiology according to previous description, (23, 24), e.g., oralfacial twitches, nodding, drooling, and unilateral forelimb clonus, were included in this study. Behavioral symptoms and ictal EEG activity were completely interrupted after 2 h of self-sustained SE by administration of pentobarbital (65 mg/kg, intraperitoneal injection).

### Lipopolysaccharide Administration

Bacterial endotoxin LPS is an endotoxin of gram-negative bacteria that is widely used to experimentally induce neuroinflammation (20, 25, 26). Naïve animals were anesthetized with isoflurane (2%) and LPS from *Escherichia coli*, serotype O26:B6 (Sigma-Aldrich, L8274, Sweden; 10 μg in 2 μl of saline) or vehicle (2 μl of saline) was stereotaxically injected into the right dorsal HPC (coordinates: 4.8 mm posterior and 5.2 mm lateral from bregma; and 6.0 mm ventral from dura, tooth bar set at −3.0 mm) using a glass microcapillary (Hamilton syringe). Animals received a single intracerebral injection of LPS or vehicle (Sal) as follows: 6 h (LPS *n* = 5 and Sal *n* = 5), 24 h (LPS *n* = 5 and Sal *n* = 5), 1 w (LPS *n* = 5 and Sal *n* = 5), and 4 w (LPS *n* = 5 and Sal *n* = 5). Six rats were used to optimize the intracerebral LPS injection to obtain appropriate and sustained microglial activation in the hippocampus (HPC). This included two different doses of LPS, 5 μg/rat (*n* = 3) and 10 μg/rat (*n* = 3). Five microgram/rat only elicited a minor microglial response in the brain. Therefore, for the purpose of this study 10 μg was selected.

### Electroencephalogram

Rats that underwent electrically-induced NCSE were continuously video- and EEG- monitored (24 h/day) throughout the experimental procedure. The EEG recordings from intra-HPC electrodes were visually evaluated and quantified in terms of number of seizures, ictal load and interictal activity (IA). Seizures were defined as ictal events on EEG lasting ≥10 s with an evolving pattern, typically consisting of an initial phase with high frequency low amplitude epileptiform activity that over time increases in amplitude and in the end develops into a lower frequency of spike-slow-wave activity (Figures 1A,B). The seizure frequency was quantified manually and the ictal load was evaluated as the total time animals exhibited seizure activity. IA was manually graded by visual evaluation according to a 0–5 scale (0 = none, 1 = <10 spikes/h, 2 = ~50 spikes/h, 3 = ~80 spikes/h, 4 = ~100 spikes/h, and 5 = more than 150 spikes/h). Animals that received LPS injection were not EEG monitored (27). All rats were subjected to 1 h of electrical stimulation in the right HPC followed by 2 h of self-sustained continuous epileptiform activity measured by EEG (Figure 1B). The semiology included non-convulsive behavior 94% of the time (Figure 1A).

### Tissue Preparation

For biochemical analyses, rats were sacrificed with an overdose of pentobarbital and decapitated. Cardiac blood was collected and the spleen was immediately removed, frozen on dry ice and stored in −80°C. Spleen samples were homogenized on ice in buffer (pH 7.6) containing (in millimolars) 50.0 Tris-HCl, 150 NaCl, 5.0 CaCl_2_, 0.02% NaN_3_, and 1% Triton X-100, and then centrifuged at 17,000 g for 30 min at 4°C. The supernatant was collected into a microcentrifuge tube, where the total protein concentration was determined by BCA protein assay (BCA, Pierce, Rockford, IL) as per manufacturer's instructions.

For immunohistochemistry, animals were transcardially perfused with 0.9% saline and 4% paraformaldehyde (PFA). Brains were post-fixed in PFA overnight, kept in 20% sucrose at 4°C for >24 h, cut in 30 μm coronal sections (10 series) on a freezing microtome, and stored in glycerol-based anti-freeze solution in −20°C.

### Immunohistochemistry

For immunohistochemistry, the following primary antibodies were used: rabbit anti-Iba1 (1:1,000, Wako, Japan) and mouse anti-CD68/ED1 (1:200, AbD Serotec, Germany). Sections were incubated with an appropriate primary antibody overnight at 4°C in blocking serum. This was followed by 2 h of incubation in secondary antibody at room temperature. For each immunohistochemical assessment, some brain sections went through the entire protocol without primary antibody incubation, to serve as the negative controls. The following secondary antibodies were used: Cy3-conjugated donkey anti-mouse/ rabbit (Jackson Immunoresearch, UK), or Alexa-488 conjugated streptavidin (1:200, Invitrogen, Sweden). The sections were mounted on gelatin-coated slides and coverslipped using a glycerol-based mounting medium (DABCO, Sigma).

### Multiplex Enzyme-Linked Immunosorbent Assay (ELISA)

Levels of IL-1β, TNF-α, IFN-γ, IL-4, IL-5, IL-6, IL-10, IL-13, and KC/GRO were measured by sandwich immunoassay methods using commercially available electrochemiluminescent detection system, plates and reagents (V-PLex Pro-inflammatory Panel 2 (rat) kit, Meso-scale Discovery (MSD), Gaithersburg, Maryland, US) as per manufacturer's instructions with minor modifications. Briefly, 100 μg (50 μl) of the protein sample (spleen/brain) and serum (2-fold dilution in 50 μl) was loaded per well in the MSD plate. The samples were incubated overnight at 4°C with shaking. For each assay, samples were analyzed in duplicates, and compared with known concentrations of protein standards. Plates were analyzed using the SECTOR Imager 2400 (Meso Scale Discovery, USA).

### Western Blot Analysis

Protein samples were denatured at 99°C for 5 min in 2x Laemmli sample buffer (Biorad, Germany). Total protein (50–100 μg) of spleen/HPC or serum (diluted 1:50) unless otherwise mentioned was resolved on precast 4–15% mini-PROTEAN TGX (Biorad) sodium dodecyl sulfate polyacrylamide gels and transferred using Trans-Blot Turbo mini nitrocellulose transfer packs (Biorad). Following this, the membranes were blocked for 2 h at room temperature in tris-buffered saline (pH 7.4) with 0.2% (w/v) Tween 20 (TBS-T) containing 5% non-fat dried milk. Membranes were then incubated overnight at 4°C with primary antibodies diluted in TBS-T containing 0.5% bovine serum albumin (BSA) (Sigma, Germany). The following primary Abs were used: mouse monoclonal anti-β actin (1:10,000, Sigma), rabbit monoclonal anti-GAPDH (1:2,000, Cell Signaling Technologies, USA), goat polyclonal anti-transferrin (1:1,000, Abcam, UK), mouse monoclonal anti-glial fibrillary acidic protein (GFAP) (1:500, Sigma), rabbit anti-S100β (1:2,000, Abcam), mouse monoclonal E-cadherin (1:500, Abcam), rabbit polyclonal anti-CD45 (1:500, Santa Cruz Biotechnology, USA), mouse anti rat-CD8 (1:200, Biorad, USA), mouse monoclonal anti-galectin-3 (1:500, Abcam), mouse anti rat CD4 (1:200, Biorad), mouse anti rat CD68 (1:500, Biorad), goal polyclonal anti-Integrin αM (1:500, Santa Cruz Biotechnology), mouse monoclonal anti-RT1B (1:500, Biorad) and rabbit anti-CX3CR1 (1:500, Abcam). After washing, membranes were incubated with secondary antibody diluted in TBS-T containing 0.5% BSA for 2 h at room temperature. Secondary antibodies used were either horseradish peroxidase-conjugated anti-mouse (1:5,000), anti-goat (1:5,000), or anti-rabbit (1:5,000) (Sigma). The membranes were then washed three times in TBS-T. Immunoreactive bands were subsequently visualized by enhanced chemiluminescence (Biorad), and images were acquired using Chemidoc XRS+ system (Biorad). Band intensities were quantified using ImageJ software (National Institutes of Health, Bethesda, Maaryland, USA). The relative protein expression was compared to the control levels and normalized by the expression of internal control transferrin for serum and β-actin or GAPDH levels for spleen and brain.

### Statistical Analysis

Statistical analyses were performed with unpaired Student's *t*-test when comparing NCSE vs. ctrls, NCSE with vs. without spontaneous seizures, and LPS vs. saline group, using GraphPad Prism software. Normal distribution of data was analyzed with histograms, DÁgostino & Pearson and Shapiro-Wilk normality tests. Significant results with parametric analyses but with skewness in the normality tests were analyzed with non-parametric Mann-Whitney test. Data are presented as means ± SEM. Differences were considered statistically significant at *p* < 0.05. Paired correlations were performed with regression analyses.

## Results

### Seizure Semiology and Electroencephalogram in Rats With Non-convulsive Status Epilepticus

During the 2 h self-sustained NCSE, the EEG pattern typically displayed rhythmic low-frequency spike and wave activity (1–3 Hz), with intermittent polyspiking and evolving frequency patterns during <10% of the time (Figure 1B). Continuous video-EEG recordings after NCSE showed that 75% of the rats that were subjected to NCSE in the 4 w survival group exhibited spontaneous seizures during week 2–4 and 25% only displayed acute symptomatic seizures within the first week. The total mean number of seizures per rat during week 2–4 measured 20 ± 14 (Figure 1C) and the mean duration 32 ± 12 min (Figure 1D). Interictal activity was evident in all rats at both 1 w and 2–4 weeks following the NCSE (Figures 1B,E). Interictal activity was evident in all rats (Figure 1B) [results previously published in Avdic et al. (7)]. Rats treated with intracerebral LPS injections were not EEG monitored in the present study. Rats with similar LPS regime have previously not exhibited epileptiform activity (27). The overall health of animals was good and no significant weight loss was observed during the experimental study.

### Lipopolysaccharide-Induced Inflammation in the Brain

We utilized unilateral intrahippocampal LPS injections to elicit an inflammatory response, based on previously performed experiments (27). We examined the microglial response in the contra- and ipsilateral hippocampus at 6 h, 24 h, 1 w, and 4 w post-LPS and it showed microglial responses in terms of increased numbers of Iba1^+^ cells and percentage of Iba1^+^/ED1^+^ cells in subregions of the HPC at all time points compared to saline injected controls (Table S1).

### Acute Transient Changes in Blood Serum Following Non-convulsive Status Epilepticus and Lipopolysaccharide Injection

**Figure 2:**
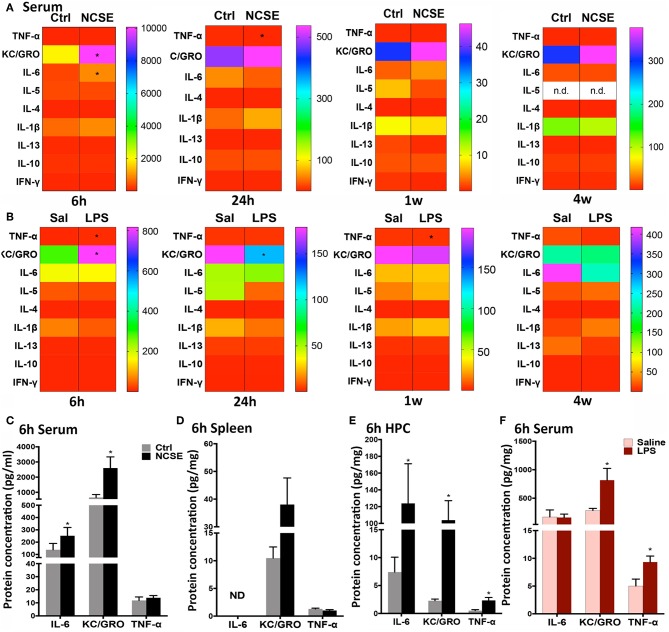
Cytokine and chemokine response following non-convulsive status epilepticus (NCSE) and LPS injection. **(A)** Heatmap plots representing mean expression level of cytokines and chemokines in serum at 6 h, 24 h, 1 w, and 4 w following NCSE and **(B)** LPS injection. The color bar on the left side in each heat map represents the non-stimulated control group (Ctrl) for NCSE and the saline-injected control group (Sal) for LPS-treated rats, respectively, and is expressed as pg/ml (serum) and pg/mg (spleen). A graphical highlight of protein expression at 6 h, when many changes occur following NCSE, of IL-6, KC/GRO and TNF-α in **(C)** serum samples, **(D)** spleen, and in **(E)** hippocampal brain tissue (HPC), and after **(F)** LPS injection in serum samples. Data are presented as mean ± standard error of mean: Ctrl; *n* = 7–12, NCSE; *n* = 7–16, Saline; *n* = 5, LPS; *n* = 5. *p < 0.05, unpaired *t*-test.

At 6 h following NCSE, ELISA analysis of common pro- and anti-inflammatory cytokines and chemokines showed increased serum levels of the pro-inflammatory cytokine interleukin (IL)-6 and chemokine keratinocyte chemoattractant/growth related oncogene (KC/GRO) compared to non-stimulated controls (Figures 2A,C). The acute NCSE-induced immune response in serum was not confirmed in spleen tissue from the same animals (Figure 2D). Instead, it correlated with increased levels in hippocampal brain tissue from the epileptic focus (Figure 2E). At 24 h post-NCSE, increased serum levels of IL-6 and KC/GRO had subsided and TNF-α levels were slightly decreased. No changes were observed at 1 or 4 w post-NCSE.

An increase in KC/GRO at 6 h after NCSE in serum was also detected after LPS-treatment, in addition to an increase in the pro-inflammatory cytokine tumor necrosis factor (TNF)-α (Figures 2A,B,F). The initial LPS-induced serum profile was no longer present at 24 h and KC/GRO levels decreased below control levels (Figure 2A). At 1 w, TNF-α was reduced and at 4 w no changes were observed. Other cytokines including IL-1β, IL-5, IL-4, IL-13, IL-10, and interferon (IFN)-γ were either unchanged or below the detection limit of the assay in serum at all time points post-NCSE and LPS (Figures 2A–C,F).

### Immune Profiles in Blood at 4 Weeks Following Non-convulsive Status Epilepticus and Lipopolysaccharide Injection

**Figure 3:**
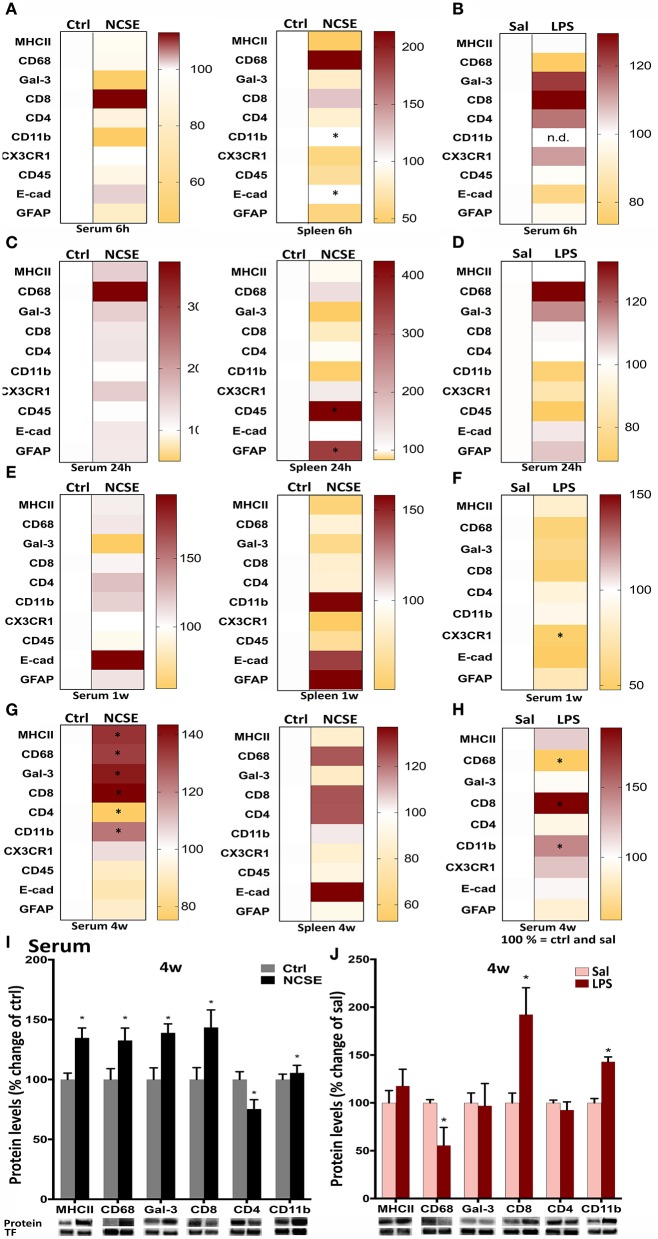
Protein levels of immune factors plotted in heat maps from NCSE rats and LPS-injected rats in serum samples and spleen 6 h, 24 h, 1 w, and 4 w after the insult. **(A–H)** Score magnitudes are shown as gradient colors from yellow to dark red, where white color bar on the left side in each heat map represents Ctrl for NCSE and saline-injected control group (Sal) for LPS-treated rats, respectively. Expression of each protein was normalized to transferrin (75 kDa) and set to 100%. Serum protein levels of MHCII (34 kDa), CD68 (110 kDa), galectin-3 (30 kDa), CD8 (34 kDa), CD4 (51 kDa), and CD11b (170 kDa) 4 w post-NCSE, and LPS injection are presented relative to their respective controls. **(I,J)** Data are presented as mean ± standard error of mean: Ctrl; *n* = 7–12, NCSE; *n* = 7–16, Sal; *n* = 5, LPS; *n* = 5. *p < 0.05, unpaired *t*-test.

Next, we analyzed other components of the adaptive and innate immune system in animals with NCSE or LPS injection. In contrast to the rapid pro-inflammatory release of cytokines and chemokines in serum at 6 and 24 h, proteins such as surface antigens (CDs) on immune cells and proteins related to phagocytosis, were not altered in serum at these early time points after NCSE (Figures 3A–D). In spleen tissue a small decrease in CD11b and E-cadherin (the latter significant with parametric analysis (*p* = 0.043) but without normal distribution of data and not significant with non-parametric analysis (*p* = 0.6), markers important for adhesion and migration (28–31), was observed at 6 h and an increase was evident in CD45 (non-parametric analysis *p* = 0.048), expressed on leukocytes (32, 33), and the astrocytic marker GFAP at 24 h post-NCSE (Figure 3C). No changes were observed in serum or spleen at 1 w post-NCSE apart from a small decrease at 1 w in chemokine receptor CX3CR1 expression (non-parametric analysis *p* = 0.008) (34, 35) post-LPS (Figure 3E). In contrast, a more substantial change was detectable for several immune factors at 4 w post-NCSE. Levels of MHCII, CD68 and galectin-3, usually associated with microglia, macrophages, and antigen presenting cells (33, 36–38) were markedly increased in serum compared to non-stimulated controls (Figures 3G,I). Levels of CD4, primarily expressed by T helper cells (39, 40), were decreased (non-parametric analysis *p* = 0.01, Figures 3G,I). These changes could not be confirmed in spleen tissue after NCSE (Figure 3G). Additionally, CD8, primarily expressed by cytotoxic T cells (41) and CD11b, present on leukocytes (42, 43) were upregulated (Figures 3G,I), while CX3CR1, CD45, E-cadherin, and GFAP were not altered at this time point (Figure 3G).

After LPS, no changes were observed in serum at 6 h, 24 h, or 1 w, except for a small decrease in chemokine receptor CX3CR1 expression (34, 35) at 1 w (Figures 3B,D,F). The increase in MHCII, CD68, and galectin-3 in serum 4 w post-NCSE was not mimicked in animals treated with LPS, where CD68 serum levels were even decreased compared to saline-injected group. However, CD8 and CD11b were upregulated after LPS-injections. CX3CR1, CD45, E-cadherin, and GFAP were not altered (Figures 3H,J).

### Immune Profile in Blood Following Non-convulsive Status Epilepticus Correlates With the Development of Subsequent Spontaneous Seizures

**Figure 4:**
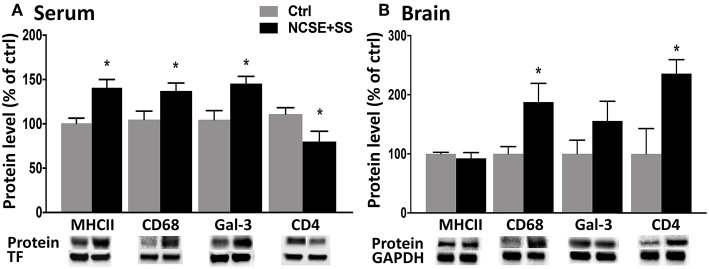
Protein levels of immune factors in serum and brain tissue from rats with NCSE with additional spontaneous seizures within 4 w post-NCSE compared to Ctrls. Representative immunoblots show MHCII (34 kDa), CD68 (110 kDA), galectin-3 (30 kDa), and CD4 (51 kDa) in **(A)** serum and **(B)** hippocampus. Bands are normalized to either transferrin (75 kDa) or glyceraldehyde-3-phosphate dehydrogenase (GAPDH; 37 kDa). Data are presented as mean ± standard error of mean: Ctrl; *n* = 5, NCSE+spontaneous seizures; *n* = 5. *p < 0.05, unpaired *t*-test.

To elucidate if the systemic changes were associated with the development of spontaneous seizures after NCSE, we divided the rats in the 4 w post-NCSE group into rats with only NCSE, and rats with NCSE that experienced spontaneous seizures (NCSE+SS) (7). Similar to the measurements shown for the entire NCSE group, cytokine and chemokine serum levels were not changed in NCSE rats with or without the development of spontaneous seizures (Figure S1). However, alterations in serum levels of MHCII, CD68, galectin-3, and CD4 seen after NCSE were only significantly altered in rats that also developed spontaneous seizures post-NCSE (Figure 4A) and not in NCSE animals without spontaneous seizures (MHCII Ctrl 100 ± 5.8 vs. NCSE 116.5 ± 16.8, CD68 Ctrl vs. NCSE 100 ± 9.0, galectin-3 Ctrl 100 ± 9.7 vs. NCSE 139.7 ± 21.5, CD4 Ctrl 100 ± 6.5 vs. NCSE 70.8 ± 16.4). Western blot analysis of hippocampal brain tissue from the epileptic focus from the same animals, showed increased expression of CD68 and CD4 in NCSE rats with spontaneous seizures, while levels of MHCII and galectin-3 remained unaltered in both groups (Figure 4B). Levels of MHCII, CD68, galectin-3, and CD4 in serum did not correlate with the total number or duration of spontaneous seizures (Table S2), nor was there a correlation between the immune profiles and the latency to develop the first spontaneous seizure (MHCII: r^2^ = 0.07, *r* = −0.27, *p* = 0.50, galectin-3: r^2^ = 0,10, *r* = −0.31, *p* = 0.42, CD68: r^2^ = 0.14, *r* = −0.38, *p* = 0.31, CD4: r^2^ = 0.04, *r* = −0.20, *p* = 0.60). The increase in CD8 observed for the entire NCSE group did not differ when subdividing NCSE rats for the occurrence of spontaneous seizures (Ctrl 100 ± 9.9 vs. NCSE 132.5 ± 9.3 vs. NCSE+SS 147.1 ± 19.4). The increase in CD11b after NCSE was, however, specific for rats with spontaneous seizures (Ctrl 100 ± 6.5 vs. NCSE 101.5 ± 5.1 vs. NCSE+SS 131.2 ± 9.1). CX3CR1 (Ctrl 100 ± 10.1 vs. NCSE 92.8 ± 4.4 vs. NCSE+SS 117 ± 21.2), CD45 (Ctrl 100 ± 11.0 vs. NCSE 86.2 ± 11.6 vs. NCSE+SS 95.3 ± 9.5), E-cadherin (Ctrl 100 ± 10.9 vs. NCSE 97.3 ± 22.6 vs. NCSE+SS 78.6 ± 8.8) and GFAP (Ctrl 100 ± 7 vs. NCSE 80.2 ± 1.9 vs. NCSE+SS 94.3 ± 4.6) remained unchanged. Also, no changes were observed in spleen tissue when subdividing the NCSE group (Figure S2).

## Discussion

**Figure 5:**
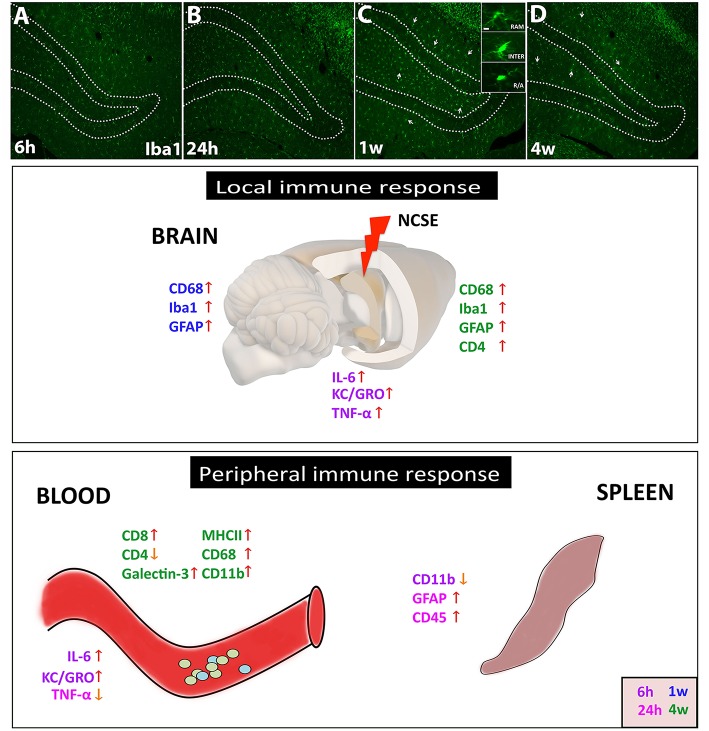
Schematic summary of the local and peripheral immune response after NCSE. **(A–D)** In the epileptic brain, one of the most common immune responses involves microglial activation, here represented in photomicrographs of Iba1^+^ cells at 6 h, 24 h, 1 w, and 4 w after NCSE. The different activation states of microglial are also represented in the inset in **(C)**, with changes in soma size and processes. The inflammatory response within the epileptic focus also includes an acute and transient release of cytokines such as IL-6, KC/GRO, and TNF-α, followed by an upregulation of microglial/macrophage and astrocytic markers at 1–4 w post-NCSE. The peripheral immune response in serum but not in spleen shares similarities with the local brain response and includes the acute transient cytokine release and a delayed increase in proteins associated with monocytes, antigen presenting cells and leukocytes. Different time-points are assigned with different colors.

Here we proved a reactive peripheral immune response following 2 h of NCSE with continuous epileptiform EEG activity and subtle behavior (illustrated in Figure 5). The immune response within the epileptic focus in the brain included an acute release of IL-6, KC/GRO, and TNF-α following NCSE and was associated with a simultaneous increase in IL-6 and KC/GRO in serum but not in spleen tissue. The increase in IL-6 was also observed after an LPS-induced reaction. Only minor transient early alterations in cell surface antigens were present in spleen tissue. At 1 w, no major changes were observed in either serum or spleen tissue, even if previous studies have shown a prominent increase in microglia and astrocyte activation within the brain at this time point (7). Instead, at 4 weeks, when a large proportion of the NCSE animals had started to develop spontaneous seizures and the brain pathology consisted of neuronal death, glial activation and changes in synaptic protein expression, a selective peripheral immune response emerged after NCSE, including altered levels of MHCII, CD68, Gal-3, and CD4. These changes were not observed in rats with NCSE without spontaneous seizures or after intracerebral LPS injection. Consequently, epilepsy development after NCSE could be associated with a delayed immune profile detected in serum.

The acute cytokine release observed in the present animal model of temporal lobe NCSE supports emerging clinical evidence of early and transiently increased levels of IL-6, IL-17A, IFN-γ, total number of leukocytes, neutrophils, and lymphocytes in plasma and serum from patients with temporal lobe epileptic seizures (10, 15, 16). More pronounced changes have also been observed in epileptic patients with hippocampal sclerosis (10). An increase in the chemokine KC/GRO was evident after NCSE and it has previously been shown to propagate neuroinflammatory responses locally by directing neutrophils to the site of injury, in addition to inducing synthesis of acute phase response cytokines such as IL-1, IL-6, and TNF-α (44, 45). However, since we observed similar acute peripheral immune response following an intracerebral LPS injection, we could not provide a seizure-specific immune response within the first 24 h post-NCSE, or show a connection between the protein alterations detected peripherally and epileptogenesis, or the development of spontaneous seizures. In addition, neither focal nor systemic increase in IL-6 levels correlated with parameters of the NCSE *per se*, e.g., frequency during NCSE (brain: r^2^ = 0.37, *r* = 0.61, *p* = 0.20, serum r^2^ = 0.0005, *r* = 0.022, *p* = 0.97).

The inflammation induced 1 and 4 w post-NCSE in rodents includes gliosis (7) with a concurrent increase in numbers of microglia with phagocytic activity (7, 46, 47). Changes in MHCII and CD68 are also well-documented after epileptic seizures and SE (48–52). The immune reaction after epileptic seizures shares similarities to the response following intracerebral LPS administration, which typically manifests with activation of microglia and production of cytokines (26, 27, 53, 54). Both insults result in long-term microglia activation for at least 7 w (26, 27, 53) or even 6 months post-SE (6). However, the peripheral immune response in the present study differed between the two insults at 4 w, at least between rats that developed spontaneous seizures after NCSE and those receiving LPS injection. Altered levels in proteins normally situated in the cell membrane of the immune cells may in the present study translate to either increased protein expression on individual cells, increased number of cells or increased soluble proteins as a result of cleavage/cell disruption. We did not register EEG activity in rats injected with LPS, but the relatively mild LPS dose used here has previously not been shown to induce abnormal EEG patterns or seizure activity (27). The occurrence of spontaneous seizures may have initiated the changes observed in antigen-presenting cell markers in serum, such as MHCII, CD68, and Gal-3. The peripheral response may then simply be the result of numbers of additional seizures. Alternatively, both seizures and the peripheral immune response are the results of the underlying emerging brain pathology during epilepsy development after NCSE. One of the mechanism by which serum immune proteins can cause increase in seizure frequency and contribute to pathology in the brain may be via oxidative stress which often accompanies inflammatory reactions, and could be a contributing factor to epilepsy pathology since it has been shown to underlie memory and mood dysfunction (55, 56). Regardless of mechanism and etiology, these findings may be of prognostic clinical value in patients after NCSE. Elevated serum levels of Gal-3 have previously been reported in patients with intractable focal epilepsy where patients continue to exhibit spontaneous seizures despite anti-epileptic drugs (57). Previous reports also suggest decreased levels of CD4^+^ T cells in patients with temporal lobe epilepsy (10) and altered levels of CD8 have been observed in experimental models of SE (13, 14).

Despite the systemic changes in cytokine and immune protein levels in serum, we found very few changes in spleen tissue with ELISA and western blot analysis. The spleen receives a rich supply of sympathetic innervation, and has lately gained attention for its pivotal role in modulating the immune response (58). The phenomenon is sometimes referred to as brain-spleen inflammatory coupling (17). Experimental studies have described dense innervation of spleen tissue with receptors on macrophages and/or lymphocytes that in turn modulate the immune response (59–62). In addition, splenectomy reduces seizure-associated mortality in rodents with convulsive SE (63). The acute transient down-regulation of CD11b may suggest increased recruitment to/from the spleen and increased levels of CD45 may reflect mobilization of leukocytes /lymphocytes at 24 h post-NCSE. Changes in GFAP level in the spleen at 24 h post-NCSE and LPS injection may reflect early astrocytic activation in the brain due to the bidirectional neuro-immune interaction.

In summary, experimental NCSE induces significant changes in the peripheral immune system and prominent changes can be detected mainly at the later stages following the insult. This distinct pathological profile in the serum is related to the subsequent brain pathology and development of spontaneous seizures, and although no conclusions can be made about the effect and origin of these alterations, the immune protein changes are only present in animals that develop epilepsy. Thus, if the immune profile in blood samples after NCSE in rats could be confirmed in humans these result may have clinical value as a predictor of subsequent epilepsy development.

## Data Availability

All datasets for this study are included in the manuscript and the Supplementary Files.

## Ethics Statement

This study was carried out in accordance with the recommendations of the regional Malmö/Lund committee for experimental animal use and the Swedish board of Agriculture (protocol number M93-14). The protocol was approved by the regional Malmö/Lund ethical committee.

## Author Contributions

CE and UA: study concept, drafting of the manuscript, study supervision, and critical revision. CE, UA, MA, and MÖ: data acquisition, data analysis, and interpretation. UA and MA: animals and surgery. All authors critically reviewed and approved the manuscript.

### Conflict of Interest Statement

The authors declare that the research was conducted in the absence of any commercial or financial relationships that could be construed as a potential conflict of interest.
