# Supplementary material for: Immune Profile in Blood Following Non-convulsive Epileptic Seizures in Rats
Source: Front Neurol. 2019 Jul 2;10:701. doi: 10.3389/fneur.2019.00701 (PMC6615316; doi:10.3389/fneur.2019.00701)
Supplement: Supplementary file 1 [file Table_1.DOCX]

**Supplementary table 1**

Number of Iba1^+^ cells and percentage of Iba1^+^/ED1^+^ cells in sub-regions of the hippocampus at different time points after lipopolysaccharide injection.

| Ipsilateral HPC | | | | | | | | | | | | | | | | | | Contralateral HPC | | | | | | | | | |
| --- | --- | --- | --- | --- | --- | --- | --- | --- | --- | --- | --- | --- | --- | --- | --- | --- | --- | --- | --- | --- | --- | --- | --- | --- | --- | --- | --- |
|  | **CA1** | | | **CA3** | | | **ML** | | | **Hilus** | | | | **CA1** | | | | **CA3** | | | | **ML** | | | **Hilus** | | |
|  | **Saline** | **LPS** | **Saline** | | **LPS** | **Saline** | | **LPS** | **Saline** | | **LPS** | | **Saline** | | **LPS** | | **Saline** | | | **LPS** | **Saline** | | **LPS** | **Saline** | | **LPS** | |
| 6h |  |  |  | |  |  | |  |  | |  | |  | |  | |  | | |  |  | |  |  | | |  |
| Iba1^+^ | 6.33±0.91 6.21±0.58 | | 4.6±0.42 5.0±0.35 | | | 39.5±3.23 44.7±2.72 | | | 43.4±2.88 54.6±3.42* | | | | 5.0±0.51 6.0±0.53 | | | | 4.23±0.46 5.27±0.36 | | | | 41.2±2.68 41.5±2.77 | | | 45.5±2.45 52.7±2.81 | | | |
| % Iba1^+^  /ED1^+^ | 0±0 8.76±3.46* | | 8.33±3.84 13.2±5.18 | | | 1.74±0.71 4.06±1.14 | | | 3.65±1.01 7.65± 0.90* | | | | 6.60± 3.64 2.08± 2.08 | | | | 3.43±2.33 10.5±6.26 | | | | 1.20±0.33 4.66±1.22* | | | 2.83±1.01 6.29±1.40 | | | |
| 24h |  |  |  | |  |  | |  |  | | |  |  | | |  |  | |  | |  | |  |  | | |  |
| Iba1^+^ | 5.7± 0.58 7.69± 1.12 | | 5.91±0.88 8.22±1.55 | | | 42.9±2.74 42.7±3.27 | | | 43.7± 2.08 53.6± 5.07 | | | | 5.92±0.67 7.00±0.62 | | | | 4.17±0.47 5.33±0.57 | | | | 38.2±2.31 45.4±3.50 | | | 41.7±2.61 44.7±2.05 | | | |
| %Iba1^+^  /ED1^+^ | 8.04±3.80 3.11±2.18 | | 41.2±17.6 70.9±24.6 | | | 7.45±2.63 20.7±6.70 | | | 15.2 ±4.57 20.8 ±6.72 | | | | 18.2±8.30 7.48±2.17 | | | | 30.2±14.1 7.68±3.45 | | | | 6.95±2.41 19.8±4.33* | | | 12.6±6.85 20.6±4.49 | | | |
| 1w |  |  |  | |  |  | |  |  | | |  |  | | |  |  | |  | |  | |  |  | | |  |
| Iba1^+^ | 2.8±0.31 3.33±0.35 | | 3.5±0.55 6.79±0.89* | | | 34.0±2.33 38.5±2.62 | | | 35.6±2.36 40.3±3.31 | | | | 3.43±0.80 3.6±0.52 | | | | 3.71±0.51 3.13±0.34 | | | | 29.6±1.47 31.5±3.35 | | | 36.5±2.24 33.8±2.90 | | | |
| % Iba1^+^  /ED1^+^ | 17.1±7.71 23.3±5.49 | | 47.4±14.8 49.2±7.73 | | | 9.63±1.80 10.8±1.52 | | | 10.4±1.92 14.3±2.82 | | | | 8.93±7.11 11.5±6.37 | | | | 15.5±5.08 29.8±9.91 | | | | 4.65±1.34 7.97±1.75 | | | 5.42±1.15 11.3±2.76 | | | |
| 4w |  |  |  | |  |  | |  |  | | |  |  | | |  |  | |  | |  | |  |  | | |  |
| Iba1^+^ | 5.27±0.67 6.36±0.77 | | 6.1±3.39 5.5±0.66 | | | 30.0±2.11 38.1±2.35* | | | 33.0±2.94 48.0±2.13* | | | | 4.75±0.51 5.08±0.40 | | | | 3.58±1.99 5.08±0.64 | | | | 28.5±2.56 34.1±2.15 | | | 36.9±3.01 44.2±3.27 | | | |
| %Iba1^+^  /ED1^+^ | 8.64±5.00 28.1±8.19 | | 24.6±10.1 61.0±9.50* | | | 5.27±1.44 8.07±1.25 | | | 15.2±3.19 25.2±2.80* | | | | 13.6±4.50 32.7±6.25* | | | | 22.9±11.3 55.9±10.5* | | | | 2.73±1.49 5.39±1.13 | | | 13.9±2.18 23.4±3.73* | | | |

**S1 Table. Microglia activation in hippocampus following LPS injection.** Number of Iba1^+^ cells and percentage of Iba1^+^/phagocytic marker ED1^+^ cells in subregions of the ipsilateral and contralateral hippocampus at different time points after intracerebral LPS injection. Data are presented as mean±standard error of mean; Saline; n = 5, LPS; n= 5. *p < 0.05, unpaired t test.
