# Supplementary material for: Immune Profile in Blood Following Non-convulsive Epileptic Seizures in Rats
Source: Front Neurol. 2019 Jul 2;10:701. doi: 10.3389/fneur.2019.00701 (PMC6615316; doi:10.3389/fneur.2019.00701)
Supplement: Supplementary file 2 [file Table_2.docx]

**Supplementary table 2.**

Correlation analysis between EEG parameters and Western blot analysis of immune proteins in the hippocampal tissue at 4wks post-NCSE

|  | MHCII | CD68 | Galectin-3 | CD4 |
| --- | --- | --- | --- | --- |
| Number of spont. seizures | r=0.11 r^2^=0.012  p= 0.81 | r=-0.27  r^2^=0.073  p= 0.556 | r=-0.61  r^2^= 0.37  p= 0.15 | r=0.080  r^2^= 0.0064  p= 0.87 |
| Total duration of seizures | r= 0.082  r^2^=0.0067  p= 0.86 | r=-0.27  r^2^= 0.073  p= 0.55 | r=-0.61  r^2^= 0.37  p= 0.14 | r=-0.061  r^2^=0.0037  p= 0.90 |
| Duration/seizure | r=-0.22  r^2^=0.049  p= 0.64 | r=-0.22  r^2^=0.048  p= 0.64 | r=0.17  r^2^=0.029  p= 0.72 | r=-0.17  r^2^=0.029  p= 0.72 |
| Interictal load | r=0.25  r^2^=0.063  p= 0.60 | r=-0.50  r^2^=0.25  p= 0.27 | r=-0.64  r^2^=0.41  p= 0.14 | r=0.43  r^2^=0.18  p= 0.35 |

**S2 Table. Regression analysis of seizure burden and protein levels.** Regression analysis of numbers, total and mean duration of spontaneous seizures and Western blot protein levels in hippocampal tissue from rats exhibiting spontaneous seizures during 4wks post-NCSE. Data are presented as Pearson’s correlation coefficient and r^2^ value. The EEG data has previously been reported in Avdic et al 2018.
