# Supplementary material for: Immune Profile in Blood Following Non-convulsive Epileptic Seizures in Rats
Source: Front Neurol. 2019 Jul 2;10:701. doi: 10.3389/fneur.2019.00701 (PMC6615316; doi:10.3389/fneur.2019.00701)
Supplement: Supplementary file 3 [file Image_1.pdf]

Supplementary figure 1

ELISA analysis of blood serum at 4wks post-NCSE

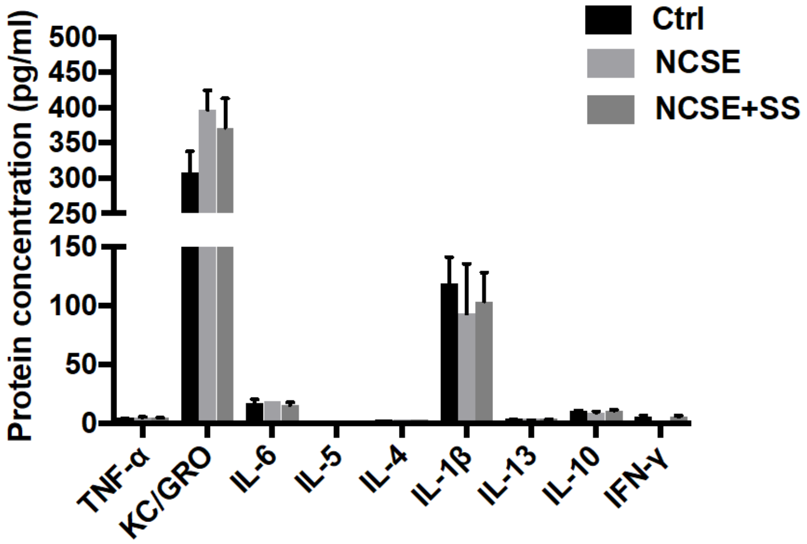

S1 Figure. ELISA analysis of pro- and anti-inflammatory cytokines and chemokines in serum 4wks following NCSE. ELISA analysis of cytokines and chemokines in serum, 4wks following NCSE with or without spontaneous seizures. Data are presented as mean±standard error of mean: Ctrl; n =7-12, NCSE; n= 3-4, NCSE+ spontaneous seizures; n=10-12. *p < 0.05, unpaired t test. TNF-α; tumour necrosis factor alpha, KC/GRO; keratinocyte chemoattractant/human growth-regulated oncogene, IL; interleukin, IFN-γ; interferon gamma.
