# Supplementary material for: Immune Profile in Blood Following Non-convulsive Epileptic Seizures in Rats
Source: Front Neurol. 2019 Jul 2;10:701. doi: 10.3389/fneur.2019.00701 (PMC6615316; doi:10.3389/fneur.2019.00701)
Supplement: Supplementary file 4 [file Image_2.pdf]

Supplementary figure 2
Western blot analysis of immune proteins in spleen tissue at 4wks post-NCSE

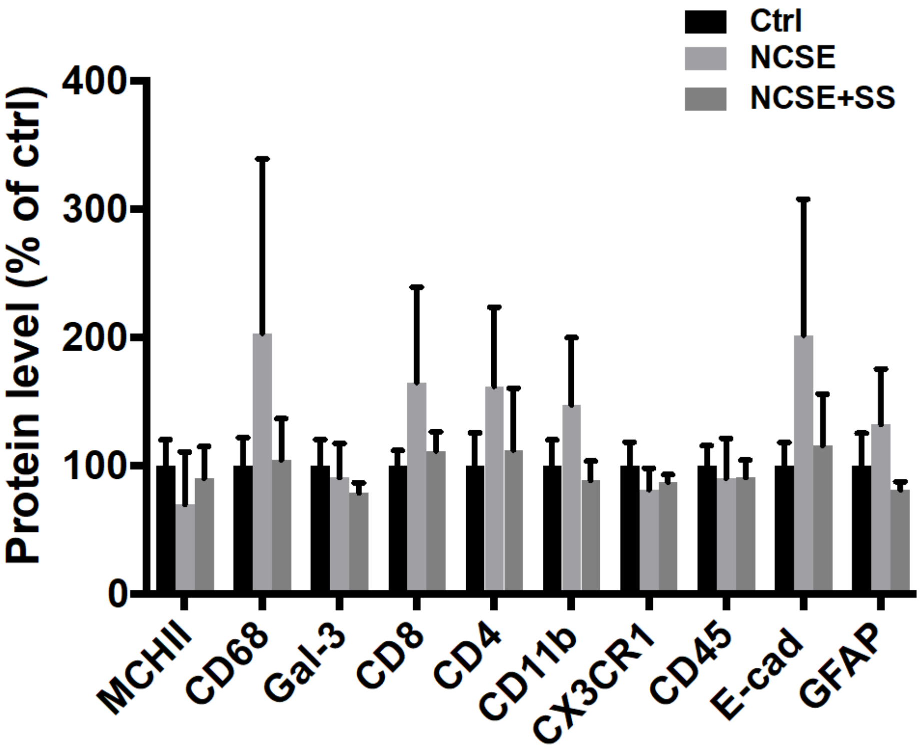

S2 Figure. Western blot analysis of spleen tissue 4wks following NCSE with or without additional spontaneous seizures. Data (mean ± standard error of mean) are presented as percentage change relative to Ctrl and normalized to either b-actin (42 kDa) or glyceraldehyde-3-phosphate dehydrogenase (37 kDa). Ctrl; n = 7-12, NCSE; n = 4, NCSE+SS; n=10-12. NCSE+SS, NCSE with spontaneous seizures; MHCII, major histocompatibility complex; Gal-3, galectin-3, E-cad, E-cadherin; GFAP, anti-glial fibrillary acidic protein. *p <0.05, unpaired t test.
